# Hyperosmotic Sisomicin Infusion: A Mouse Model for Hearing Loss

**DOI:** 10.21203/rs.3.rs-4096027/v1

**Published:** 2024-04-01

**Authors:** Ayse Maraslioglu-Sperber, Fabian Blanc, Stefan Heller, Nesrine Benkafadar

**Affiliations:** Stanford University School of Medicine; Stanford University School of Medicine; Stanford University School of Medicine; Stanford University School of Medicine

**Keywords:** Cochlea, Hair cell, Supporting cell, Hearing loss, Sisomicin

## Abstract

Hearing impairment arises from the loss of either type of cochlear sensory hair cells. Inner hair cells act as primary sound transducers, while outer hair cells enhance sound-induced vibrations within the organ of Corti. Established models, such as systemic administration of ototoxic aminoglycosides, yield inconsistent and variable hair cell death in mice. Overcoming this limitation, we developed a method involving surgical delivery of a hyperosmotic sisomicin solution into the posterior semicircular canal of adult mice. This procedure induced rapid and synchronous apoptotic demise of outer hair cells within 14 hours, leading to irreversible hearing loss. The combination of sisomicin and hyperosmotic stress caused consistent and synergistic ototoxic damage. Inner hair cells remained intact until three days post-treatment, after which deterioration in structure and number was observed, culminating in cell loss by day seven. This robust animal model provides a valuable tool for otoregenerative research, facilitating single-cell and omics-based studies toward exploring preclinical therapeutic strategies.

## Introduction

A primary cause of hearing impairment is the loss of outer hair cells, which is irreversible in mammals. It is challenging to induce hair cell loss in mice reliably. Noise exposure or systemic application of aminoglycosides typically results in an incomplete loss of outer hair cells that is pronounced at the base of the cochlea and becomes sparser towards the apex (Cho et al, 2013; Fernandes & Lin, 2014; Fernandez et al, 2020; Hirose & Sato, 2011; Jansen et al, 2013; Ju et al, 2017; Maraslioglu-Sperber *et al,* 2024; Oesterle et al, 2008; Schmitz et al, 2014; Taylor et al, 2008; Wu et al, 2001).

To overcome the limitations of acoustic overstimulation and systemic aminoglycoside exposure, several transgenically engineered mouse strains have been developed that allow for a more clinical and, most significantly, virtually complete ablation of all cochlear hair cells (discussed by (Maraslioglu-Sperber *et al,* 2024)). The most established of these ablation models is the *Pou4f3*^*DTR/+*^ mouse (Golub et al, 2012; Tong et al, 2015). Here, the human diphtheria toxin receptor gene *(HBEGF)* is expressed in a hair cell-specific manner, which allows elimination of inner and outer hair cells with a single injection of diphtheria toxin (Konishi et al, 2017; Lee et al, 2021; Tong et al., 2015). We argue that this method is excellent and clinical, but it might not reflect the physiological complexity caused by pathologically relevant ototoxins, such as aminoglycosides. Existing aminoglycoside-induced hair cell loss *in vivo* models, however, require repeated systemic injections over a few days, resulting in asynchronous and incomplete outer hair cell loss (Chen & Saunders, 1983; Jiang et al, 2005; Wu et al., 2001). A single high dose of aminoglycoside, in conjunction with loop diuretics given over a few days, can lead to the complete elimination of outer hair cells (Fernandes & Lin, 2014; Jansen et al., 2013; Taylor et al., 2008). Still, the dosing must be carefully controlled to prevent mortality (Fernandes & Lin, 2014). Moreover, all these protocols result in asynchronous hair cell loss, extending between forty-eight hours (Taylor et al., 2008) and four weeks (Jansen et al., 2013), and the low-frequency locations tend to be more resistant (Ju et al., 2017; Oesterle et al., 2008).

We reason that for single-cell -omic profiling technologies to investigate ototoxic and otoregenerative responses of inner ear cells systematically (Benkafadar et al, 2021; Benkafadar et al, 2024; Janesick et al, 2022; Matsunaga et al, 2020), it will be important to develop alternative methods to eliminate hair cells in a controlled manner in adult mice. We consider the *Pou4f3*^*DTR/+*^ mouse as an excellent model for this purpose. Here, we aimed to develop a comparable method for the adult mouse cochlea based on a pathologically relevant ototoxic insult.

A single sisomicin infusion into the chicken ear’s lateral semicircular canal eliminates all auditory hair cells in the avian hearing organ, the basilar papilla (Benkafadar et al., 2021; Janesick A, 2022). This method resulted in a highly reproducible time course of hair cell demise and response in surrounding cells, such as the supporting cells that engage in a regenerative response. It enabled single-cell RNA-sequencing of hair cells and supporting cells at specific time points post-sisomicin infusion and revealed gene expression changes in response to aminoglycosides and hair cell loss (Benkafadar et al., 2021; Benkafadar et al., 2024; Janesick et al., 2022). Despite the massive hair cell loss in response to sisomicin infusion, the avian inner ear can fully recover with complete hair cell regeneration, proper innervation, and restoration of hearing thresholds within five weeks (Sato et al, 2024). We hypothesize that establishing a similar insult method in adult mice will allow the future application of single-cell -omics methods for direct cross-species comparison between mammals and birds.

Here, we established a robust model of acute outer hair cell loss in adult mice by administering a hyperosmotic sisomicin solution (ho-sisomicin) into the posterior semicircular canal of four to five-week-old FVB mice. We determined that outer hair cells underwent apoptotic death within a narrow time window of three to eleven hours post-treatment, demonstrating a synchronized pattern of demise. We also noted a protracted loss of inner hair cells starting at three days post-treatment. Supporting cells remained generally unaffected. Hair cell loss was accompanied by a permanent absence of auditory thresholds observed from three hours post-infusion, based on measuring auditory brainstem responses (ABRs) and distortion product otoacoustic emissions (DPOAEs).

## Results

### Drug infusion into the inner ear

**Figure 1:**
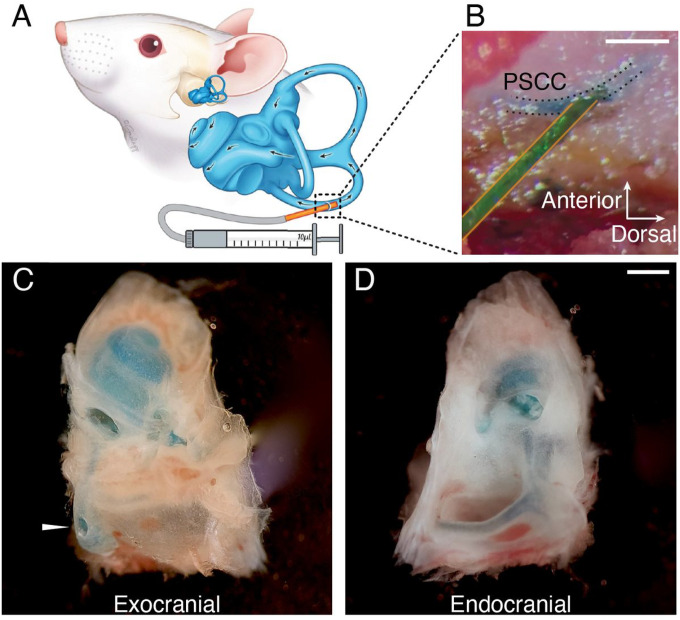
Infusion into the Posterior Semicircular Canal. **A.** Schematic representation of the posterior semicircular canal (PSCC) infusion installation, with a small polyimide tube (orange) inserted into the bony PSCC lumen connected to a 10 μL syringe and micropump. B. Operative microscope view during the infusion of methylene blue-stained buffer solution into the PSCC. **C, D.** Temporal bone dissection immediately after injection, demonstrating diffusion of the solution throughout the inner ear. The injection site is indicated by the white arrowhead in C. Blue staining is observed in the semicircular canals and all cochlear turns. Scale bars = 500 μm.

We investigated the diffusion of buffer solution into the inner ear of four to five-week-old mice by infusing it into the posterior semicircular canal (Fig. 1A, B). Methylene blue was used to visualize the extent of the solution’s diffusion throughout the inner ear. After injection, the animals were immediately sacrificed, and the temporal bones were dissected (Fig. 1C, D). We found that 1.1 – 1.5 μL of infused buffer was sufficient for obtaining a distribution of the solution through all turns of the cochlea and the semicircular canals. We concluded that infusion into the posterior semicircular canal facilitates drug diffusion throughout the inner ear, including the full tonotopic range of the cochlea.

### Sisomicin treatment and serendipitous discovery of the effects of hyperosmolality on hair cells and supporting cells

We next infused the aminoglycoside sisomicin into the left semicircular canal of postnatal day (P) 28 mice. Sisomicin offers high purity, assuring consistent and reliable experimental outcomes. It is a minor constituent of the multi-componential gentamicin complex and displays a high level of ototoxicity by selectively targeting sensory hair cells (Grahek & Zupancic-Kralj, 2009; Huth et al, 2015; Kitasato et al, 1990; O’Sullivan et al, 2020), allowing controlled induction of hair cell loss. The rapid and substantial hair cell loss caused by sisomicin is advantageous for time-sensitive experiments and studying acute hair cell damage effects (Benkafadar et al., 2021).

**Figure 2:**
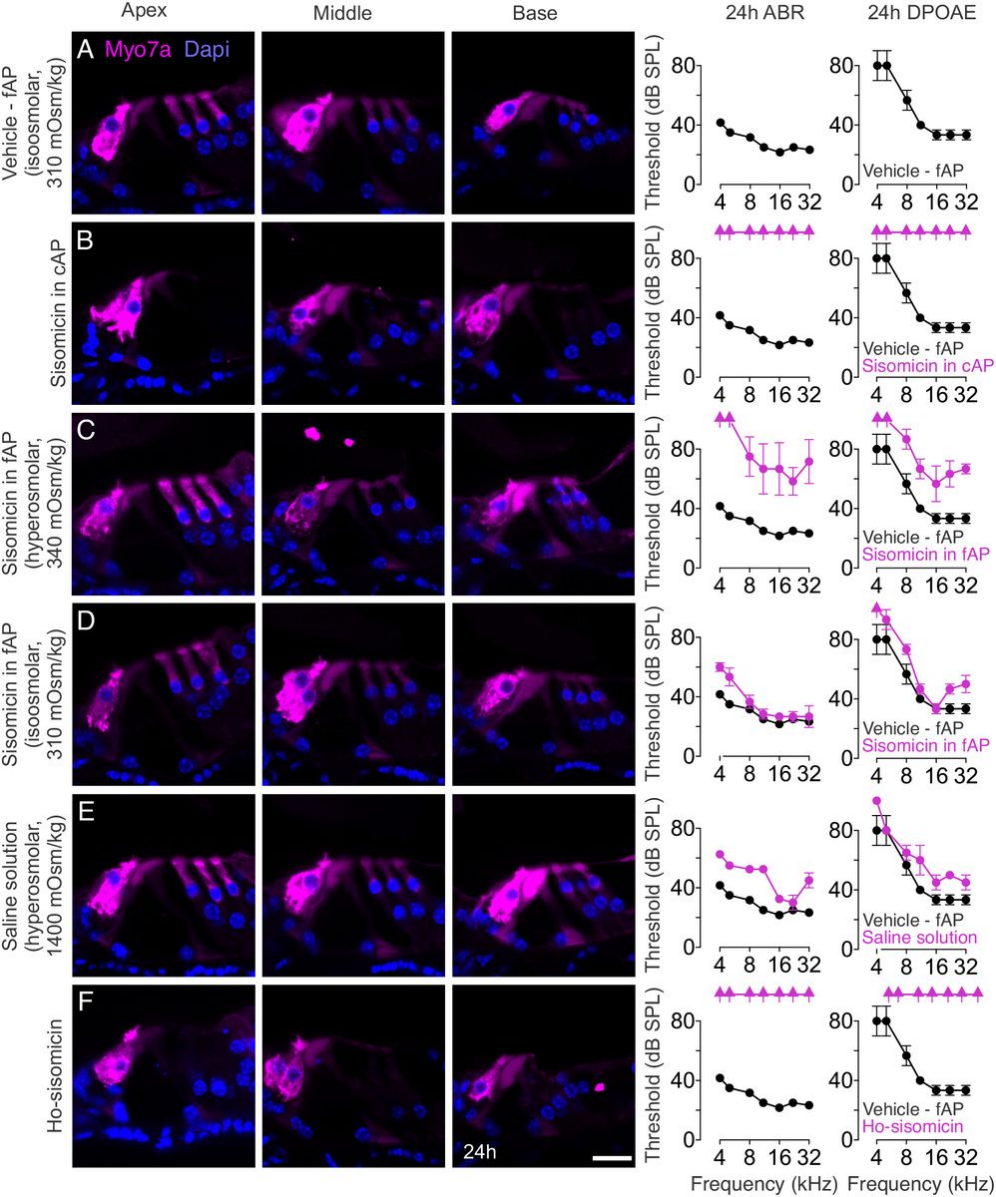
Synergy of Hyper-Osmolality and Sisomicin. **A.** 24 hours after infusion of freshly prepared artificial perilymph (fAP) (vehicle fAP control), normal histology (no hair cell loss) is apparent in transverse vibratome sections from the apex, middle, and base. We measured normal ABR and DPOAE thresholds (black). **B.** Infusion of 20 mg/mL sisomicin in commercially procured artificial perilymph (cAP) resulted in the complete loss of outer hair cells from apex to base, accompanied by a loss of ABR and DPOAE thresholds (magenta), compared with vehicle fAP control measurements. **C.** Infusion of 20 mg/mL sisomicin in fAP, not adjusted for osmolality and therefore slightly hyperosmolar. A few outer hair cells in the base were lost after 24 h, accompanied by a shift of ABR and DPOAE thresholds (magenta). **D.** 20 mg/mL sisomicin in fAP with adjusted osmolality elicited minimal ABR threshold shifts and no hair cell loss (magenta). **E.** Injection of hyperosmotic saline solution resulted in a few missing outer hair cells in the basal turn and a slight ABR and DPOAE threshold shift (magenta). **F.** The combination of 20 mg/mL sisomicin and hyperosmotic saline solution resulted in complete outer hair cell loss in the base and middle parts of the cochlea, with some remaining outer hair cells in the apex, and no detectable ABR and DPOAE thresholds (magenta). ABR and DPOAE thresholds of the vehicle fAP control cochlea are shown in black in panels B - F for reference. Scale bar = 20 μm; N = 3 – 5 cochleae in each group.

To ensure the infusion procedure itself did not cause damage, we used a vehicle injection of freshly prepared artificial perilymph (fAP) or physiological saline solution and observed no hair cell loss, with mice displaying normal ABR and DPOAE thresholds 24 hours after injection (Fig. 2A, Suppl Fig. 1A). This confirmed the safety of the infusion procedure, consistent with previous reports (Kawamoto et al, 2001; Nakagawa et al, 2003; Suzuki et al, 2017) and uninjected control ears (Suppl. Figure 1B).

To maintain consistency and minimize variability, we decided to use a commercial preparation of artificial perilymph (cAP). Infusing sisomicin in cAP resulted in complete outer hair cell loss and no detectable ABR or DPOAE thresholds at 24 hours post-treatment (Fig. 2B). Concurrently, we conducted a control experiment using sisomicin in freshly made artificial perilymph (fAP), where we observed no hair cell loss but a noticeable shift in ABR and DPOAE thresholds (Fig. 2C).

Puzzled by this inconsistency, we contacted the manufacturer for details about the cAP preparation but obtained unsatisfactory responses. To investigate further, we measured the osmolality of the 1X cAP and discovered it to be extremely hyperosmotic at approximately 5,000 mOsm/kg. Becoming aware of the possible importance of infusion buffer osmolality (Choi & Oghalai, 2008), we proceeded to measure the osmolality of the 20 mg/mL sisomicin in fAP which revealed a mild hyperosmolarity (340 mOsm/kg) (Fig. 2C). Subsequently, we carefully prepared an iso-osmolar solution of 20 mg/mL sisomicin in fAP (310 mOsm/kg). This adjusted preparation caused only a small ABR threshold shift at low frequencies and did not induce any hair cell loss (Fig. 2D).

Our next objective was to determine whether osmolality shift alone or the combined effect of both factors was essential for inducing outer hair cell depletion. To investigate this, we chose 0.9% normal saline, which by itself did not cause hair cell loss or affect hearing fidelity (Chen & Fechter, 2003) (Suppl. Figure 1A). We hypothesized that saline presents the advantage of having the simplest composition of solutions for inner ear infusion. Infusing a 1,400 mOsm/kg hyperosmolar saline solution resulted in small ABR and DPOAE threshold shifts and loss of a few outer hair cells in the basal turn of the cochlea (Fig. 2E). Infusion of 20 mg/mL sisomicin in a 500 mOsm/kg hyperosmolar saline solution produced a similar result (Suppl. Figure 1C). Finally, the infusion of 20 mg/mL sisomicin diluted in saline solution to achieve a final osmolality of 1,400 mOsm/kg (ho-sisomicin), replicated the results of the infusion of 20 mg/mL sisomicin in cAP It led to an almost complete outer hair cell loss (Fig. 2F), and an absence of ABR and DPOAE thresholds (Fig. 2F), suggesting that a combination of high osmolality and sisomicin induces profound outer hair cell death and hearing loss.

**Figure 3:**
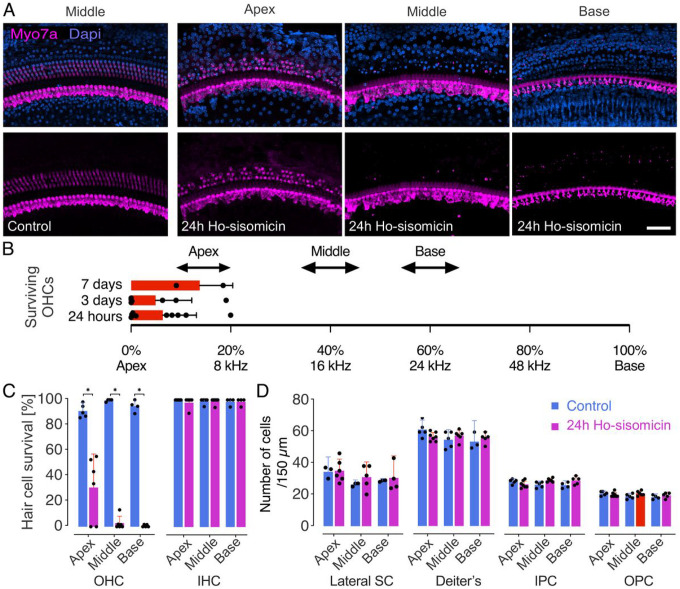
Ho-sisomicin Treatment Ablates Outer Hair Cells after 24 hours. **A.** Wholemounts labeled with antibodies to Myo7a to reveal inner and outer hair cells. The right ear control cochlea was unaffected by ho-sisomicin infusion into the left ear’s posterior semicircular canal. Three rows of outer hair cells and a single row of inner hair cells were visible in all turns; the middle section is shown. At 24 hours post-ho-sisomicin treatment (PhoST), all outer hair cells in the base and middle were ablated; some animals showed surviving outer hair cells in the apex. Inner hair cells were not affected. **B.** Surviving outer hair cells (OHCs) are located in the most apical region of the cochlea. This was consistent after 24 hours, 3 days, and 7 days PhoST. Each dot represents a different cochlea PhoST and is located at the transition between the region of partial surviving OHCs and the more basal complete OHCs loss. The arrowed lines represent the location of the sections shown in (A). **C.** Quantification of surviving hair cells 24 hours post-infusion revealed a significant loss of OHCs. Inner hair cells (IHC) were not affected. **D.** We counted supporting cell nuclei in the different subtypes of cochlear supporting cells, which we identified by their distinct location and cytomorphology. This analysis revealed no significant difference between the PhoST and control groups. Bar plots show the mean with standard deviation as whiskers. Each dot represents a single count from a 200 μm portion of the apex, middle, or base of one cochlea. Abbreviations: SC - supporting cells; IPC - inner pillar cells; OPC - outer pillar cells. Scale bar = 100 μm; N = ≥ 4 cochleae in each group (control + experimental). Wilcoxon rank-sum tests, *p* < 0.05 (*).

Whole-mount immunohistochemistry of the cochlea confirmed an almost complete loss of outer hair cells at 24 hours post-treatment with ho-sisomicin (Fig. 3A). None of the assessed animals had more than 20% surviving outer hair cells in the extreme apical region, and 44% of animals exhibited complete outer hair cell loss (Fig. 3B and Suppl. Figure 2A). Quantitative analysis revealed a significant reduction in the number of surviving outer hair cells, primarily concentrated in the middle and basal regions of the cochlea (Fig. 3C). We did not detect significant differences in the numbers of inner hair cells and the various subtypes of cochlear supporting cells between the ho-sisomicin solution and control groups at 24 hours post-treatment (Fig. 3C, D and Suppl. Figure 2A, B).

These results suggest a synergistic effect between sisomicin and hyperosmolality in specifically targeting the outer hair cells without an immediate effect on inner hair cells and supporting cells.

### Time course of ho-sisomicin-induced outer hair cell death

**Figure 4:**
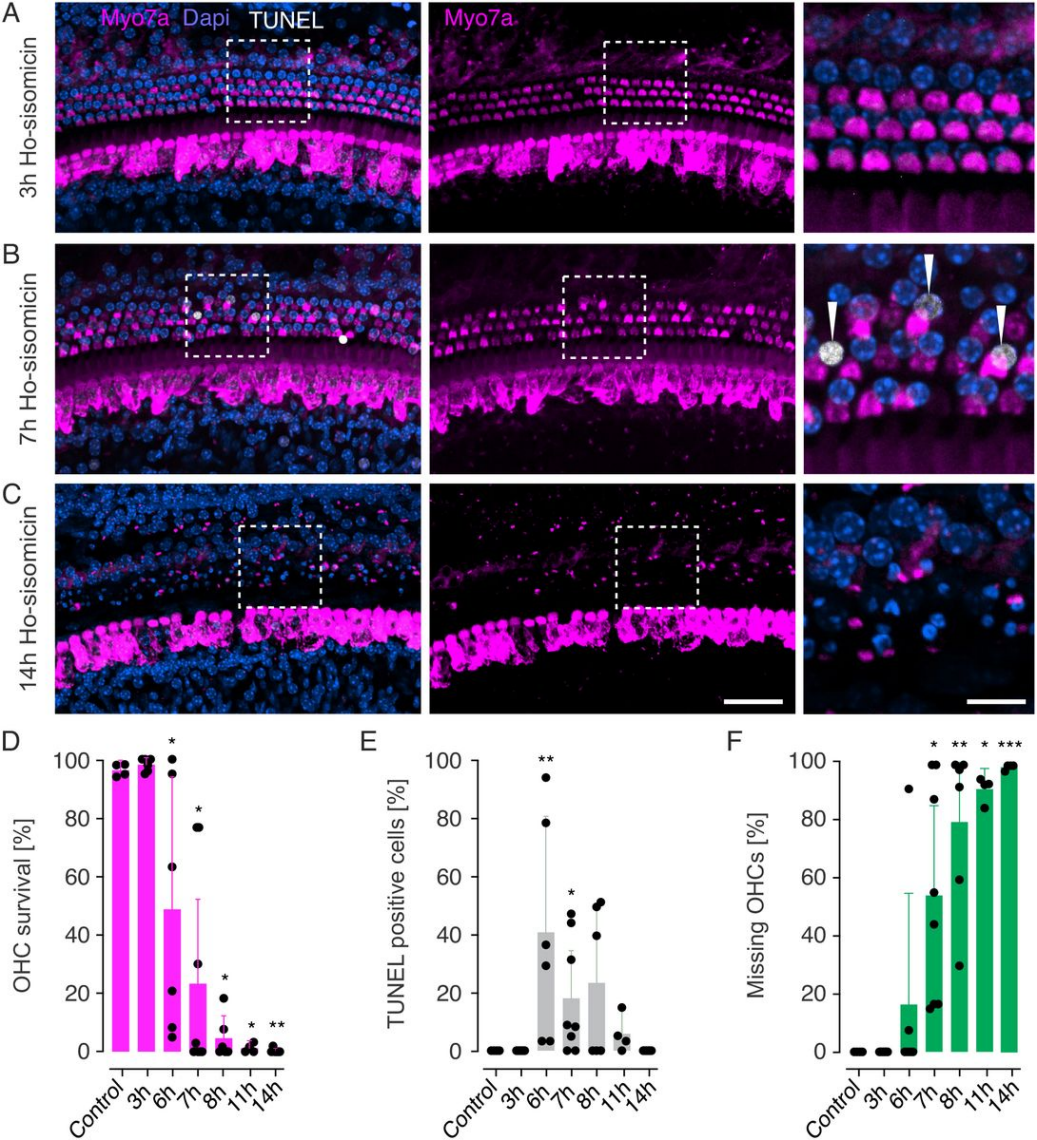
Rapid Outer Hair Cell Death via Apoptosis. **A.** At 3 hours post ho-sisomicin treatment (PhoST), the cochlear sensory epithelium shows normal morphology without TUNEL-positive nuclei. **B.** TUNEL-positive outer hair cell nuclei were visible at 7 hours PhoST, while no TUNEL-positive supporting cell or inner hair cell nuclei were detected. **C.** At 14 hours PhoST, complete outer hair cell loss with surviving inner hair cells and supporting cells was observed. Some Myosin 7a staining remained in the outer hair cell region, associated with hair cell debris. The examples shown in A-C are from a middle region of the cochlear duct. **D.** Quantification of surviving outer hair cells (OHC) shows a significant decrease starting at 6 hours PhoST, with complete OHC loss observed at 11 −14 hours PhoST. **E.** Quantification of TUNEL-positive OHC nuclei shows that dying hair cells are in late-stage apoptosis from 6h −11h PhoST. The majority of TUNEL-positive OHCs was observed at 6 hours PhoST. **F.** Gradual increase in missing OHCs or OHC corpses from 6 hours PhoST onward. Bar plots show the mean with standard deviation as whiskers. Each dot represents a count from a 200 μm portion of the middle or base of one cochlea. Left scale bar = 50 μm, right scale bar: 10 μm; N = 3 – 6 animals for each time point. Dunn’s test for multiple comparison, N = 3 – 6 animals for each time point. *p* < 0.05 (*), *p* < 0.001 (**), *p* < 0.001 (***).

We next performed experiments to investigate the time course and the mechanism of ho-sisomicin-induced outer hair cell death at different time points post-treatment using terminal deoxynucleotidyl transferase dUTP nick end labeling (TUNEL). At 3 hours post-ho-sisomicin treatment, no TUNEL-positive nuclei were observed, and the sensory epithelium appeared normal (Fig. 4A). However, at 7 hours post-ho-sisomicin treatment, we detected TUNEL-positive outer hair cell nuclei while no TUNEL-positive inner hair cell and supporting cell nuclei were visible (Fig. 4B). TUNEL detects nuclear DNA fragmentation, which is a hallmark of apoptosis, and it indicates that the labeled cells are in the final stages of programmed cell death. By 14 hours post-ho-sisomicin treatment, we observed a complete loss of outer hair cells with surviving inner hair cells and supporting cells (Fig. 4C). Some Myosin 7a immunoreactivity, likely associated with outer hair cell debris, remained in the outer hair cell region (Fig. 4C).

Quantification confirmed the histological observations and revealed a significant decrease of surviving outer hair cells at 6 hours post-ho-sisomicin treatment and complete outer hair cell loss after 14 hours (Fig. 4D). TUNEL-positive outer hair cell nuclei peaked at 6 hours post-treatment, indicating that most of the cells enter the late stages of apoptotic cell death between 3 and 6 hours after exposure to ho-sisomicin. DNA fragmentation in outer hair cells was evident between 6- and 11- hours post treatment (Fig. 4E). Moreover, the number of missing outer hair cells and outer hair cell debris increased gradually, starting from 6 hours post-ho-sisomicin treatment (Fig. 4F).

Our results indicate that ho-sisomicin treatment induces a rapid and selective death of outer hair cells through apoptosis. In contrast, inner hair cells and supporting cells appear unaffected within the first 24 hours.

### Delayed inner hair cell death

Previous studies have demonstrated that aminoglycosides preferentially target outer hair cells, and inner hair cells generally remain unaffected (Forge & Schacht, 2000; Jansen et al., 2013; Oesterle et al., 2008). Consistent with these findings, we observed no significant changes in the number of inner hair cells 24 hours after ho-sisomicin injection, despite the near-complete loss of outer hair cells (Fig. 3A, C).

**Figure 5:**
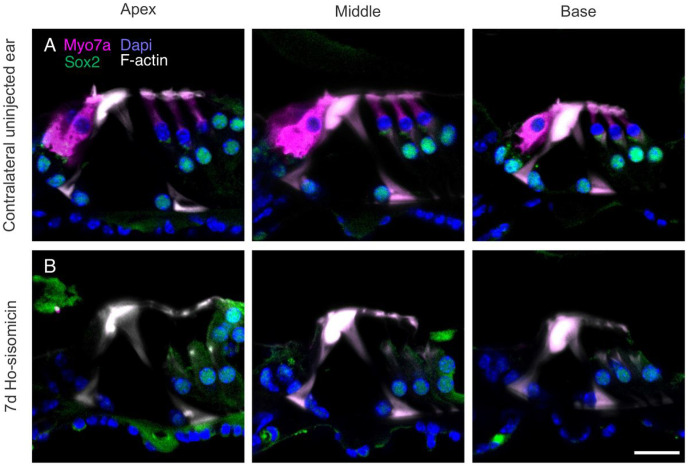
Profound Inner Hair Cell Loss 7 Days Post-ho-sisomicin Infusion. **A.** Right ear control cochlea at 7 days PhoST, showing the presence of three rows of outer hair cells and a single row of inner hair cells in magenta (Myosin 7a). **B.** In the ho-sisomicin infused left cochlea, all inner hair cells were lost at 7 days PhoST. The F-actin-rich pillar cells remained unaffected. Scale bar = 20 μm.

To explore possible long-term effects, we examined the fate of inner hair cells beyond the initial 24-hour post-treatment period and waited for seven days after ho-sisomicin infusion. While the contralateral right uninjected ear showed no damage at seven days (Fig. 5A), over 70% of ho-sisomicin-infused cochleae displayed a consistent and complete loss of inner hair cells (Fig. 5B). We did not encounter animals without inner hair cell loss but rather with a more extensive cell loss. For example, on some occasions, the delayed damage of the organ of Corti after seven days included supporting cells (Suppl. Figure 3A). Moreover, one animal exhibited a flat epithelium seven days after the ho-sisomicin infusion (Suppl. Figure 3B). These observations emphasize the inherent *in vivo* variability that needs to be acknowledged as a limitation of the method employed in our study. An evaluation of the individual surgical infusions suggests that the main cause of variability could be the placement of the infusion needle and different levels of leakage during drug administration. With the delivered volume set to a specific parameter, such as 1.1 3bL, the ultimate volume that is being delivered into the posterior semicircular canal in each surgery is likely lower than the volume extruded by the micropump. We estimate that each experimenter will ultimately have a sweet spot that defines the average absolute infused volume of the drug solution in each experiment.

**Figure 6:**
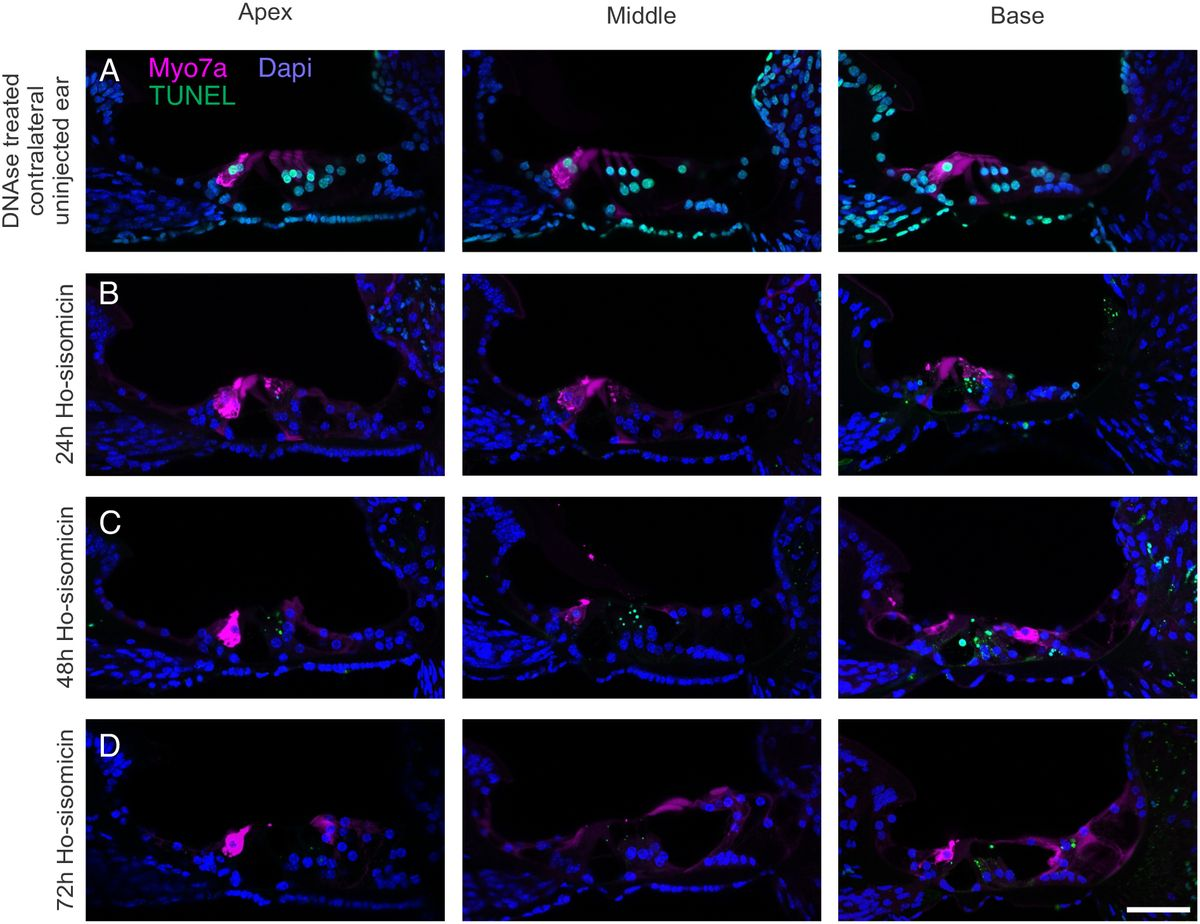
Ho-sisomicin-Induced Inner Hair Cell Death is not Caused by Apoptosis. **A.** DNAse treated contralateral uninjected ear shows normal cochlear epithelium morphology with three rows of outer hair cells and a single row of inner hair cells in magenta. A brief treatment with DNAse was used to create fragmented DNA as a positive control for TUNEL (green nuclei). **B, C.** 24 hours and 48 hours post ho-sisomicin treatment, inner hair cells were not affected. Some TUNEL-positive outer hair cell debris is visible. **D.** At 72h post-injection, inner hair cell death was visible. The remaining inner hair cells have TUNEL-negative nuclei. Scale bar = 50 μm; N = 3 cochleae at each time point.

We wanted to understand further when and how inner hair cells die. To do so, we subjected the samples to TUNEL at 24, 48, and 72 hours post ho-sisomicin infusion. We treated the contralateral uninjected ear with DNAse to serve as a technical control for TUNEL (Fig. 6A). At 24 and 48 hours post ho-sisomicin infusion, all examined cochleae displayed intact inner hair cells with normal cytomorphology (Fig. 6B, C, and Suppl. Figure 4A). However, at 72 hours post-infusion, we observed some loss of inner hair cells (Fig. 6D and Suppl. Figure 4B). Interestingly, we did not detect TUNEL-positive inner hair cell nuclei at any investigated time point (Fig. 6 and Suppl. Figure 4). These results suggest that the delayed loss of inner hair cells within the observed time frame is not associated with apoptosis.

Our findings unveil that combining sisomicin with hyperosmolarity results in a relatively consistent impact on inner hair cells resulting in their death, albeit delayed with respect to outer hair cell loss. Furthermore, our study demonstrates that this phenomenon is not limited to inner hair cells alone but also involves the potential loss of supporting cells in certain instances. The observed inconsistent and asynchronous cellular demise underscores the intricate nature of this process within an *in vivo* context.

### Structural changes in the stria vascularis

**Figure 7:**
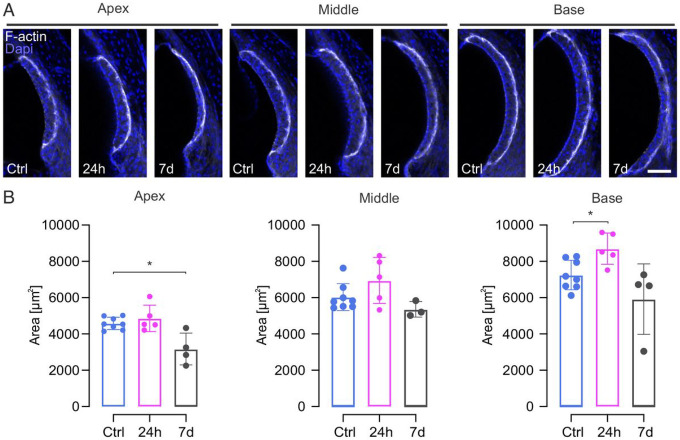
Structural Changes in the *Stria Vascularis* after Ho-Sisomicin Infusion. **A.** Vibratome sections of controls and cochleae 24h and 7 days post ho-sisomicin infusion. Cell nuclei and F-actin are fluorescently labeled with Dapi and phalloidin. **B.** Quantification of the stria’s cross-sectional area revealed a mild swelling of the stria in the base and middle regions of the cochlea after 24h. Seven days post ho-sisomicin treatment, we detected a significant decrease of the stria’s cross-sectional areas. Bar plots show the mean with the standard deviation as whiskers. Scale bar = 50 μm; N = 3 – 8 cochleae at each time point. Dunn’s test for multiple comparisons, *p* < 0.05 (*).

Ototoxic trauma, particularly noise but also aminoglycosides (Hirose & Sato, 2011; Taylor et al., 2008; Wang et al, 2002) does not only affect the organ of Corti but also other cochlear structures. The *stria vascularis* is a physiologically essential structure in the lateral wall of the cochlea. Its function is ion homeostasis and generation of the endocochlear potential in the *scala media*. We measured the cross-sectional area of the *stria vascularis* at 24 hours and seven days post-ho-sisomicin treatment, comparing it with the contralateral uninjected ear (Fig. 7). The cross-sectional area was determined through F-actin labeling of the tissue that revealed the stria’s histomorphology (Fig. 7A). Quantification indicated a significant increase of the cross-sectional area in the basal part of the cochlea 24 hours post-infusion. Seven days following the infusion, a notable reduction was observed, with significance confined to the apical region (Fig. 7B).

Our findings suggest that the damage induced by ho-sisomicin extends beyond the cochlear epithelium and also impacts the *stria vascularis*.

### Ho-sisomicin treatment causes permanent hearing loss

**Figure 8:**
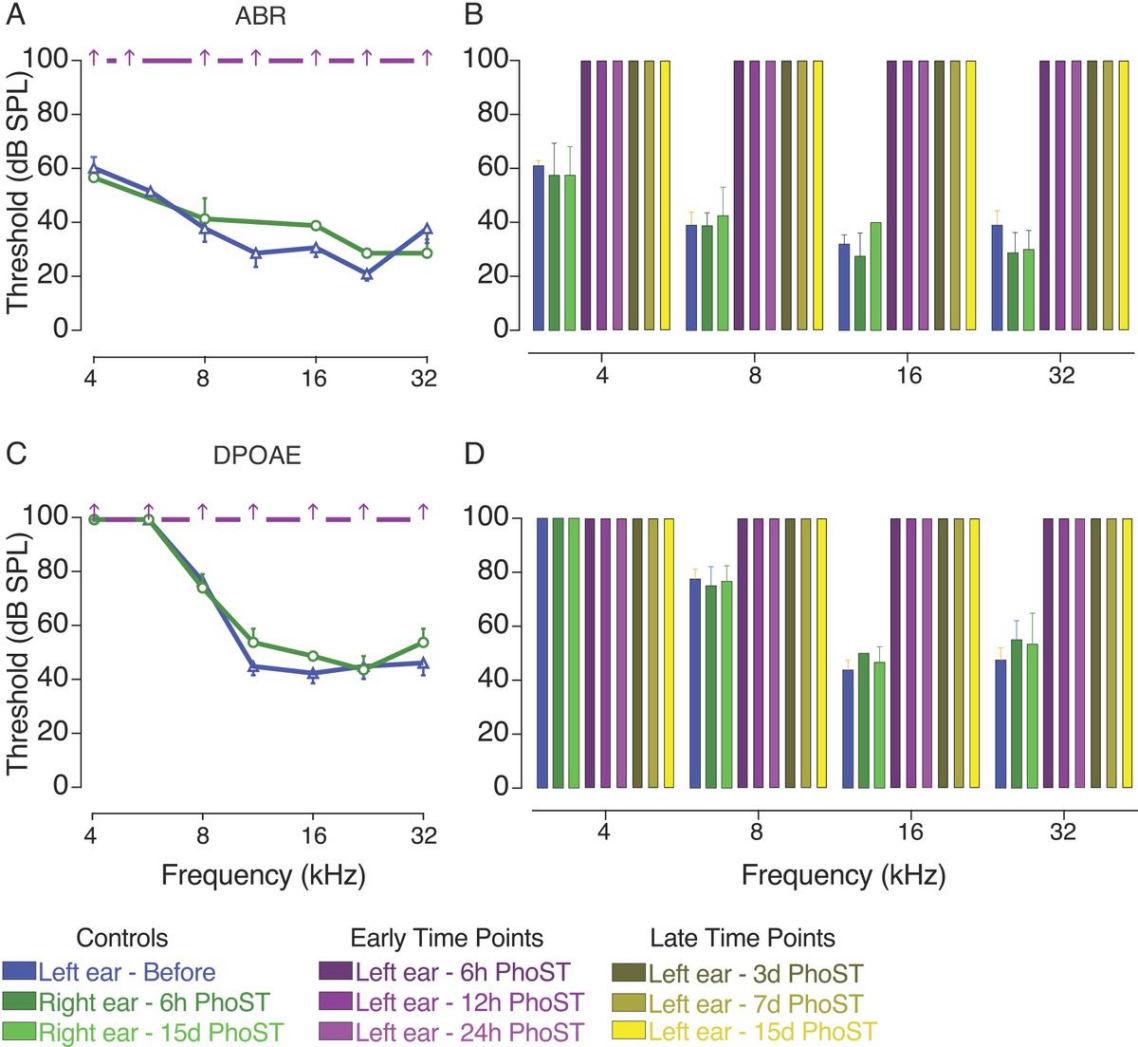
Ho-sisomicin Treatment Induces Rapid Hearing Loss. **A.** Auditory Brainstem Response (ABR) thresholds in pre-infusion control (blue) and contralateral post-infusion control (dark green) inner ears show normal hearing thresholds. Six hours post-ho-sisomicin treatment (PhoST, dark magenta), no ABR thresholds were detectable. **B.** Controls and additional time points reveal the permanent loss of ABR thresholds. **C.** Distortion Products of Otoacoustic Emissions (DPOAE) thresholds obtained before ho-sisomicin treatment (blue) or from contralateral controls (dark green) show normal DPOAE thresholds. No thresholds were detected six hours PhoST. **D.** Controls and additional time points reveal the permanent loss of DPOAE thresholds. Bar plots show the mean with the standard deviation as whiskers. N = 3 – 4 animals at each time point. Dunn’s test for multiple comparison, all threshold losses (ABR and DPOAE) at all time points were highly significant (*p* < 0.001).

The administration of ho-sisomicin significantly impacted hearing, leading to rapid and irreversible hearing loss. Six hours post-treatment, we observed a complete absence of ABR and DPOAE thresholds across all frequencies in injected left ears (Fig. 8). The ABR waveforms recorded from the affected left ears showed prolonged latency and reduced amplitude exceeding 70 dB SPL, accompanied by signal crosstalk from the contralateral ear (Ding et al, 2020). However, following surgical destruction of the right ear, these ABR waveforms disappeared, and no signals above the noise amplitude were recorded up to 100 dB SPL. We classified the 100 dB SPL thresholds as “no threshold” values for the tested frequencies in the left ear.

Thus, our findings support the notion that ho-sisomicin infusion into the posterior semicircular canal enables effective drug diffusion throughout the cochlea, resulting in rapid apoptotic outer hair cell loss accompanied by immediate and permanent hearing loss.

## Discussion

Hearing loss affects hundreds of millions of individuals worldwide, necessitating the development of novel therapeutic interventions to prevent or even restore cellular loss and cochlear function in patients. The loss of cochlear hair cells is the major primary contributor to permanent hearing loss. To effectively develop treatments, it is crucial to have animal models in which hair cell loss can be induced reliably. Hair cells are the most sensitive component of the mammalian cochlea and can be eliminated with acoustic overstimulation or ototoxic drugs such as aminoglycoside antibiotics. Despite this susceptibility, ablating hair cells in the mouse cochlea using loud noise or systemic administration of aminoglycosides is a challenging endeavor (Maraslioglu-Sperber *et al.,* 2024). The shortcomings of many existing drug-based damage models are likely owed to the fact that the effective drug concentration inside the cochlear duct is insufficient to kill the more drug-resistant apical outer hair cells and inner hair cells.

We sought to overcome these shortcomings. First, we replaced the often-used aminoglycoside gentamicin - a natural product and a mixture of different isoforms and impurities - with the chemically defined sisomicin (Benkafadar et al., 2021). Sisomicin is a highly ototoxic impurity of gentamicin (O’Sullivan et al., 2020). Second, we decided to deliver sisomicin directly into the inner ear *via* the posterior semicircular canal, a direct route into the inner ear (Kawamoto et al., 2001; Suzuki et al., 2017). We previously used this strategy to efficiently ablate auditory hair cells in the chicken inner ear (Benkafadar et al., 2021; Benkafadar et al., 2024; Janesick et al., 2022).

We surmised that using artificial perilymph for drug delivery would replicate a physiological environment that closely resembles the natural conditions in the inner ear, allowing for an accurate assessment of sisomicin’s impact on hair cells and associated structures. To ensure consistency and minimize variability, we opted to utilize a commercial preparation of AP for our sisomicin infusion experiments. Our first results of infusing sisomicin in this commercial AP preparation resulted in a rapid and complete loss of outer hair cells and the absence of ABR and DPOAE thresholds. This result was confusing because when we infused sisomicin in freshly prepared AP or saline, we detected noticeable shifts in ABR and DPOAE thresholds without accompanying hair cell loss.

To investigate the factors contributing to this discrepancy, we analyzed the properties of the commercial AP preparation and discovered that it had an extremely high osmolality despite the distributor’s assurance that it was a 1X ready-to-use buffer solution. This prompted us to carefully prepare an iso-osmolar solution of sisomicin in AP aiming to match the physiological osmolality of extracellular fluids. Remarkably, the iso-osmolar sisomicin solution resulted in minimal shifts in ABR thresholds and no hair cell loss.

Further experiments were conducted to elucidate the essential factors responsible for the depletion of outer hair cells: sisomicin alone, osmolality shift alone, saline versus fAP or the combined effect of sisomicin and hyperosmotic buffer. Infusion of hyperosmolar saline or sisomicin in saline without adjusting osmolality resulted in partial loss of outer hair cells and noticeable shifts in ABR and DPOAE thresholds. Importantly, when sisomicin was infused in hyperosmolar saline, it replicated the results observed with sisomicin infusion in commercial AP leading to a nearly complete loss of outer hair cells and the absence of ABR and DPOAE thresholds. These findings suggested a synergistic potentiation of hyperosmolality and sisomicin. This could be attributed to a comparable mechanism seen in mouse damage models using systemic aminoglycoside injection, where coadministration of loop diuretics enhanced the ototoxicity (Taylor et al., 2008).

Analysis of cochlear whole-mounts confirmed the extensive loss of outer hair cells after ho-sisomicin treatment. Intriguingly, inner hair cells and supporting cells remained unaffected within the first 24 hours, suggesting an initial selectivity of ho-sisomicin for outer hair cells. This selective impact aligns with previous studies demonstrating the preferential targeting of outer hair cells by aminoglycosides.

Time course experiments provided insights into the rapid and selective death of outer hair cells through apoptosis. About 40% of outer hair cells were TUNEL-positive within six hours of ho-sisomicin infusion. Because DNA fragmentation is a marker of the latest stage of apoptotic cell death, we assume that induction of apoptosis in outer hair cells happens very fast after the infusion, within the first six hours. The loss of hair cells was sudden and extensive, with evidence suggesting a minimal role for macrophages or supporting cells in immediate debris clearance. This dramatic cell loss contrasts with the gradual decline seen in aging or with milder ototoxic events.

Remarkably, delayed loss of inner hair cells was observed between days 3 and 7 post-ho-sisomicin infusion without corresponding apoptotic indicators. These findings suggest the involvement of different mechanisms in the demise of inner and outer hair cells, warranting further investigation.

Examination of the sensory epithelium seven days after ho-sisomicin infusion revealed the complete loss of inner hair cells. We detected some variability in a small proportion of specimens that manifested in more severe damage, such as supporting cell loss or even epithelial flattening. It is important to note that while variability exists, the method generally is reliable and results in virtually complete ablation of all cochlear hair cells after seven days.

The absence of ABR and DPOAE thresholds across all frequencies indicates an immediate and profound impact on auditory function already three hours post-ho-sisomicin infusion. If only outer hair cells were initially impacted by the infusion, we would observe an absence of DPOAE thresholds but a moderate ABR threshold shift. However, the immediate absence of thresholds on both evaluations was detected before the death of outer or inner hair cells. This observation suggests that the compromise of cochlear function is more complex than simply causing the death of hair cells.

Despite these open questions, we argue that the method has an applied advantage: its synchronicity and the uniformity of its impact across the entire cochlea. As we have previously shown for the avian inner ear (Benkafadar et al., 2021; Benkafadar et al., 2024; Janesick et al., 2022), we expect that the synchronicity, and especially the two distinct waves of outer hair cell loss followed by inner hair cell demise, will be useful to determine gene expression changes in dying hair cells and surviving cochlear floor cells. Moreover, the more complex cochlear pathology after ho-sisomicin presents an useful comparative model when investigated parallel to the more clinical hair cell elimination that can be achieved with diphtheria toxin in the well-established *Pou4f3*^*DTR/+*^ mouse model. Both mouse models are excellent platforms for future single-cell omics experiments to determine a useful baseline for developing cochlear cell reprogramming therapies toward hair cell regeneration or similar endeavors.

## Methods and Materials

### Animal surgery

Male and female FVB mice aged 4 to 5 weeks (Charles River Laboratories, St-Constant, QC) were used. The Stanford University Institutional Animal Care and Use Committee approved the housing and animal procedures. All methods were performed in accordance with the relevant guidelines and regulations, including adherence to the principles outlined in the ARRIVE guidelines.

Mice were anesthetized with 2 L/min oxygen and 2% isoflurane using a nose cone. During the surgery, the body temperature of the mice was maintained at 37.5°C utilizing a heating pad. The postauricular area was shaved, and the skin was disinfected using alternating 10% povidone-iodine (Betadine, Purdue Products, Stamford, CT, Cat #301013-OC) followed by 70% ethanol for three times. The mice received subcutaneous administration of 300 μL of carprofen (5 mg/kg, Rimadyl, Zoetis, Parsippany, NJ) for analgesia. The animals were confirmed to exhibit no reflex to painful stimulation before surgery.

Under sterile conditions, a left post-auricular incision was made. The sterno-cleïdo-mastoïd muscle was bluntly dissected, exposing the posterior part of the temporal bone and the square angle formed by the lateral and posterior semicircular canals. A canalostomy of the posterior semicircular canal was performed using a 26G beveled needle. Effusion of lymph confirmed the opening of the endosteum of the bony canal. The hole was then widened using a micro-driller (Performance Micro Tool, Cat #100M2X300S) to approximately the size of the outer diameter of the polyimide tube (124 μm, Microlumen, 039).

The procedure involved inserting a polyimide tube into the posterior semicircular canal, which was sealed to the temporal bone using two drops of tissue adhesive (3M Vetbond, St. Paul, MN) to prevent leakage. The polyimide tube was attached to a PVC tube (Tygon) connected to a 10 μL Hamilton syringe. A total of 1 1 μL of sisomicin or control solution (for details, see “Drug preparation”) was infused into the posterior semicircular canal at a rate of 4 nL/s using a Micropump (UMP3 Micro 4, World Precision Instruments).

After the injection, the tube was left in place and was closed by melting the free tip with an electrocautery pen (Thermo Fisher Scientific, Cat #NC9721806). Special care was taken to avoid any leakage at the injection site during and after the injection. The skin was closed in two layers with surgical glue. The total duration of the procedure was approximately 30 minutes. The isoflurane was reduced to 0% for recovery, and pure oxygen was provided at 4 L/min on a heating pad until the animal moved normally. After the injection, the animals were monitored daily. They presented moderate signs of left vestibular deficit (head tilt, circling behavior) during the first two days after injection. These symptoms were compatible with normal activity and feeding and improved in the following days.

### Drug preparation

The sisomicin solution was prepared by dissolving sisomicin (MedChemExpress, Cat #HY-B1222) at a concentration of 20 g/L in sterile 0.9% sodium chloride solution or artificial perilymph. To increase the solution’s osmolality, mannitol (Sigma-Aldrich, Cat #M4125–100G) was added and thoroughly mixed until completely dissolved. The amount of mannitol required to achieve the desired osmolality varied depending on the initial osmolality of the sisomicin solution and the desired final osmolality. Two types of artificial perilymph were utilized in this study. The first type was purchased from Biochemazon (Cat #BZ285), while the second type was prepared in-house in accordance with a previously described method (George et al, 2021).

A dose-response curve was used to determine the appropriate osmolality of the sisomicin solution to achieve the desired hair cell damage. High osmolality sisomicin (ho-sisomicin) was freshly prepared immediately before each injection for a group of 3 animals.

To ensure the accuracy and validity of our results, we included control groups in our study. The first control group underwent a sham treatment, which involved an infusion of sterile 0.9% sodium chloride solution or artificial perilymph without adding sisomicin. The second control group received a dose of iso-osmolar sisomicin solution. These control groups were essential for comparison and establishing the specific effects of the hyperosmotic sisomicin solution used in our experimental group.

### Functional hearing assessment

To assess the effect of the ho-sisomicin infusion on the function of hair cells, we conducted ABR and DPOAE recordings before and at different time points after surgery (6 hours, 14 hours, 24 hours, three days, seven days, and 15 days). All functional assessments were performed under anesthesia using ketamine (100 mg/kg) and xylazine (10 mg/kg) in a Faraday-shielded, anechoic, sound-proof chamber. We maintained the body temperature at 37.5°C with the help of a heating pad.

### Distortion Product of Otoacoustic Emissions

DPOAEs were recorded in the external auditory canal using a calibrated probe. The two primary tones of frequency f1 and f2 were generated with a constant f2/f1 ratio of 1.2, geometrically centered around the tested frequency, and the distortion product 2f1-f2 was processed by the RZ6 Processor and visualized by BioSigRZ software (Tucker-Davis Technologies). The f1 and f2 were presented simultaneously, and the ratio remained the same, from 80 dB SPL to 20 dB SPL in 10 dB decrements. We tested 4, 5.7, 8, 11.3, 16, 22.6, and 32 kHz. For each tested frequency, the threshold was defined as the lowest intensity where a distortion product 2f1-f2 emerged more than 20% over the neighboring noise.

### Auditory Brainstem Responses

Auditory Brainstem responses were recorded using the RZ6 Processor and BioSigRZ software (Tucker-Davis Technologies). Electrodes were placed subcutaneously on the vertex, beneath the ear, and in the leg of anesthetized mice, connected to a RA4PA preamplifier and RA4LI head stage. Tone bursts of 10 ms duration were delivered at a rate of 21/s at 4, 5.7, 8, 11.3, 16, 22.6, and 32 kHz. Sounds were provided by a Tucker-Davis loudspeaker in a calibrated closed-field condition. Amplification of responses from the electrodes was achieved with a Nexus type 2690 amplifier (Bruel and Kjaer) and averaged 512 times. Level-amplitude functions of the ABRs were obtained at each frequency by varying the level of the tone bursts from 0 to 80 dB SPL in 5 dB decremental steps. ABR threshold was defined as the lowest sound level at which a reproducible waveform could be observed. For the right ear, after the initial complete set of frequencies recorded before injection, only four frequencies (4, 8, 16, 32 kHz) were tested to assess the contralateral effect of the left ear injection.

### Dissection of Sensory Organs and Vibratome Sectioning

The heads were bisected and the bony ear capsules were removed using surgical scissors (Cat #F.S.T. 14002–13). The tissue was then fixed in 4% paraformaldehyde in PBS overnight at 4°C, following which the cochleae were either briefly stored in PBS at 4°C or directly processed. For vibratome sections, cochleae were decalcified in 0.5 M EDTA (Thermo Fisher Scientific, Cat # AM9261) for 3 to 4 days. To maintain tissue structure during sectioning, the cochleae were embedded in 4% low melt agarose in disposable molds (Cat #VWR 15160–215). Embedded specimens were stored at 4°C in a humidified chamber. The cochleae were sectioned transversely using a Leica VT1200 vibratome (100 μm thickness, 1 mm amplitude, 0.6 mm/sec speed) in cold PBS. The resulting sections were decanted onto a Sylgard^®^ 184 silicone plate, and excess agarose was removed by punching out round cochlear sections with a 1.5 mm biopsy punch.

### Immunohistochemistry

Immunocytochemistry was performed in cochlear whole-mount preparations and on anatomical vibratome sections. We used anti-Myosin 7a (1/500, Proteus Biosciences, Cat #25–6790) and Sox-2 (1/50, Santa Cruz Biotechnology, Cat #sc-365823). Rhodamine-conjugated phalloidin (1/2000, Thermo Fisher Scientific, Cat #A22287) was used to label F-actin. Secondary antibodies were diluted at 1:500. These included donkey anti-mouse and anti-rabbit, respectively conjugated to Alexa 488 and Alexa 568 (Thermo Fisher Scientific, Cat #A-21202 and Cat #A10040). DNA was stained with Dapi (1/1000 MilliporeSigma, Cat #D3571). DeadEnd^™^ fluorometric TUNEL System was used to identify apoptotic DNA fragmentation (Promega, Cat #G3250). DNAse was used as a positive control to demonstrate the efficacy of the TUNEL staining in the cochlear epithelium. After labeling, we mounted the sections on glass slides in antifade medium (Citifluor CFM-3; Electron Microscopy Sciences, Cat #17979–30) with a 0.12 mm spacer (ThermoFisher, Cat #S24735) between the slide and the coverslip. We conducted primary antibody omission controls to ensure that secondary antibodies did not produce specific labeling. All experiments were performed at least in triplicate, and representative images are shown.

### Imaging and Quantitative Analysis

Confocal microscopy was performed using a Zeiss LSM700 microscope at 1.0 zoom and 20X or 40X magnification (Plan-Apochromat, Numerical Aperture 1.3, oil immersion), and images were acquired using Zen Black software. To analyze the confocal stacks and generate maximum intensity projections, ImageJ software (NIH) was used. To quantify the number of cells per independent experiment, at least three randomly selected 100×100 μm quadrants from the cochlea’s basal, middle, and apical regions were imaged. Cell counts for cells labeled with Myosin 7A and Sox2 antibodies were performed in these images using the Cell Counter plug-in for Fiji software. At least three independent experiments were performed for each data point to ensure the accuracy and reproducibility of the results.

### Quantification and Statistical Analysis

Cell counts were exported and graphically displayed in GraphPad Prism software. Significant differences between groups were assessed with one-way ANOVA (Kruskal-Wallis test by ranks); once the significance of the group differences (*p* ≤ 0.05) was established, Dunn’s tests were used for post-hoc comparisons between pairs of groups. *p*-values are indicated in the legends of each figure.

## Data Availability

All data generated or analyzed during this study are included in this published article and its Supplementary Information. The source data are provided with the paper.
